# “When They See a Wheelchair, They’ve Not Even Seen Me”—Factors Shaping the Experience of Disability Stigma and Discrimination in Kenya

**DOI:** 10.3390/ijerph18084272

**Published:** 2021-04-17

**Authors:** Giulia Barbareschi, Mark T. Carew, Elizabeth Aderonke Johnson, Norah Kopi, Catherine Holloway

**Affiliations:** 1 UCL Interaction Centre & Global Disability Innovation Hub, University College London, London WC1E 6BT, UK; c.holloway@ucl.ac.uk; 2 Policy, Influencing and Research, Leonard Cheshire, London SW8 1RL, UK; mark.carew@leonardcheshire.org (M.T.C.); elizabeth.johnson@leonardcheshire.org (E.A.J.); 3 Shujaaz Inc., Nairobi 00502, Kenya; norah.kopi@shujaazinc.com

**Keywords:** assistive technology, disability, Kenya, stereotypes, stigma

## Abstract

Disability stigma in many low- and middle-income countries represents one of the most pervasive barriers preventing people with disabilities from accessing equal rights and opportunities, including the uptake of available assistive technology (AT). Previous studies have rarely examined how disability stigma may be shaped through factors endemic to social interactions, including how the use of assistive technology itself may precipitate or alleviate disability stigma. Through two strands of work, we address this gap. Via a series of focus groups with Kenyans without disabilities (Study 1) and secondary data analysis of consultations with Kenyans with disabilities and their allies (Study 2), we identify shared and divergent understandings of what shapes disability stigma and discrimination. Specifically, Kenyans with and without disabilities were cognizant of how religious/spiritual interpretations of disability, conceptions of impairments as “different” from the norm, and social stereotypes about (dis)ability shaped the experience of stigma and discrimination. Moreover, both groups highlighted assistive technology as an influential factor that served to identify or “mark” someone as having a disability. However, whereas participants without disabilities saw assistive technology purely as an enabler to overcome stigma, participants with disabilities also noted that, in some cases, use of assistive technologies would attract stigma from others.

## 1. Introduction

Stigmatizing attitudes and beliefs towards disability represent one of the most pervasive and complex barriers that limits access to health care, education, employment, civic rights and opportunities for socialization for people with disabilities [1,2,3]. The damaging impact of disability stigma is widely acknowledged and, according to article 8 of the UN Convention on the Rights of Persons with disabilities, developing strategies, campaigns, policies and other initiatives to combat disability stigma and ensure that all people with disabilities are treated with dignity and respect is also a duty of the 182 countries who ratified the treaty [4]. Although the majority of literature focused on understanding disability stigma has been carried out in high-income settings [5,6,7], in the last decade, an increasing number of scholars have conducted studies looking at the negative stereotypes, prejudices and inaccurate beliefs that shape disability stigma in the Global South [3,8,9,10]. Most of these studies have described how these stigmatizing beliefs are often driven by a combination of personal and societal factors, ranging from misconceptions concerning the causes of different impairments (e.g., disability to be seen as a form of curse or punishment); assumptions about the lack of capabilities of people with disabilities; or discriminatory practices that actively endorse separation between people with and without disabilities [3,9,11,12]. Yet, there is a dearth of comparative studies that examine the perspectives of both people with and without disabilities of disability stigma and discrimination, including how the use of assistive technology may shape stigmatizing interactions.

Goffman described stigma as a social phenomenon in which an individual is labeled by society on the basis of a single attribute that is judged as non-desirable [13]. As a result of this negative label, the person is discredited and rejected by the rest of society. In turn, this negatively affects one’s behavior and self-identity, creating a gap between virtual and actual social identity [13]. This conceptualization makes it clear that stigma in general, and disability stigma in particular, is not a unidirectional phenomenon that develops in an isolated state in the minds of the “normal” individuals in mainstream society cascading down on the affected victims [14]. Instead, stigma is socially constructed through a series of endemic interactions between people with and without disabilities which are shaped by personal and societal misconceptions that are often bilateral [15,16,17,18,19]. This conceptualization of stigma as an interactional phenomenon “*based on multiple grounds, including prejudice, religious beliefs, low expectations and even fear*” was also outlined in the 2019 Report of the Office of the United Nations High Commissioner for Human Rights on Awareness-raising under article 8 of the Convention on the Rights of Persons with Disabilities [20], which recognizes the importance of understanding how disability stigma develops in order to design effective strategies to combat prejudice and discrimination. Yet, despite the intrinsic dual nature of stigma as a social and interactional construct, most empirical research carried out in this area aims at either evaluating the impact of stigma on people with disabilities [21,22,23] or developing and testing stigma-reduction strategies among people without disabilities [10,24,25,26,27]. Even when researchers have focused on identifying the main factors that shape stereotypes and stigma surrounding disabilities, this is usually from solely the perspective of people without disabilities [28,29,30,31,32,33] or, less commonly, looking at the construction of self-stigma among people with disabilities [34,35,36,37]. The aim of this paper is to bridge the gap between these two perspectives by presenting two studies side by side that respectively investigated how disability is conceptualized by young people without disabilities and how stereotypes and stigma are perceived by people with disabilities and their allies. In our analysis, we focus particularly on overlapping and diverging beliefs to understand the root of mismatched interactions that contribute to the construction of stigma. We do this within the context of assistive technology, which adds to the contextual experience of people with disabilities.

Emerging research carried out in the Global North has also shown how contextual factors, which are largely linked people’s individual experiences and the way social interactions between people with and without disability unfold, have a substantial effect on the choices surrounding assistive technologies (ATs) [38,39,40]. For example, it is not uncommon for people with disabilities to choose a mainstream device over a more traditional AT, which might be better suited to the performance of a specific function, in order to stand out less in a group of people [38]. In more extreme circumstances, an individual might choose to not use or use an assistive technology in order to not be judged as “too disabled” or “not disabled enough” by others, rather than basing their choice solely on the capabilities they feel are available to them in different situations [40]. Similarly, a diary study carried out by Shinohara and Wobbrock showed that people without disabilities often evaluated different aspects of ATs, such as their appearance, their functionality and the context in which they were used, to form opinions and make judgements about disability [41]. Moreover, recent experimental work suggests that the presence of assistive devices may modify the stereotypical judgments that people without disabilities confer on people with disabilities (e.g., of (in)competence) [42].

Amidst this small body of research, an aspect that is largely overlooked in existing research is understanding the role that assistive technology (AT) plays in shaping disability stigma in the Global South, including in Kenya. According to estimates from the WHO, 9 out of 10 individuals who need AT do not have access to them, equating to over 1 billion people in the world, 80% of whom live in the Global South [43,44]. A recent scoping review on barriers to AT access identified both disability stigma and discrimination as significant obstacles that could obstruct the uptake of AT, even when these are available and affordable [45]. Finally, research carried out with wheelchair users and providers in Kenya has shown that, although the functionality of ATs is important, considerations around identity, self-expression and presentation might play an even bigger role in AT choices [46]. In this paper, we explore the experiences and perceptions that Kenyans with and without disabilities have of AT, including perceptions of how AT impacts on disability stigma and discrimination.

Specifically, we believe that developing effective and inclusive strategies to tackle misconceptions about disabilities and reduce discrimination is only possible if we build a thorough understanding of how stigmatizing beliefs are built through everyday interactions between people with and without disabilities. Furthermore, it is essential to consider how these interactions are shaped by the unique context in which they take place. To this end, in this paper, we seek to identify the factors that influence, both positively and negatively, the interactions between Kenyan people with and without disabilities and how these factors contribute to disability stigma and discrimination. We also explore how Kenyan people with and without disabilities perceive AT and how AT impacts on their experience of disability stigma and discrimination. Crucially, across two studies, we examine the experiences of Kenyans with and without disabilities to identify overlapping and diverging beliefs to understand the root of misunderstandings in intergroup interactions that contribute to the construction of stigma.

Both studies seek to address the following research questions:

1. What are the factors that shape stigmatizing beliefs and interactions between Kenyans with and without disabilities?
2. What is the role of AT that shapes stigmatizing beliefs and interactions between Kenyan with and without disabilities?

## 2. Materials and Methods

### 2.1. Context of the Studies

Kenya is one of the most influential countries in the East African region, rated by the OECD as a ‘low- and middle-income’ country, and with an estimated population of 51 million [47]. In the last decade, Kenya has witnessed an incredibly fast development in the ICT sector, with Nairobi often being referred to as the “Silicon Savannah”, which has raised the country’s international profile [48]. Increased investment in the digital industry has boosted the national economy and created a strong culture of entrepreneurial activity [49]. Yet, despite these advances, the inequality rate remains high according to UN indices [50]. Data on prevalence of disability are highly conflicting. The 2019 housing census [51] shows a national prevalence rate of disability of 2.2%, whereas data from the World Report on disability estimate a 15.2% prevalence rate [44]. Physical disabilities were found to be the most prevalent followed by visual, hearing and communication [51]. Regardless of impairment type, almost half of people with disabilities in Kenya are unemployed and 67% of people with disabilities, compared to 52% of people without disabilities, reported living below the poverty line [52,53].

### 2.2. Study 1: Conceptualization of Disability and Assistive Technologies among Kenyan Youth without Disabilities

#### 2.2.1. Participants and Procedures

The data collected and analyzed for this study were generated by the Ground Truth for the project on developing innovative strategies to reduce disability and AT stigma among youth in Kenya conducted by Shujaaz Inc. as part of the AT2030—Life Changing Assistive Technology for All funded by the UK Foreign and Commonwealth Development Office (FCDO) and led by the Global Disability Innovation Hub. The aim of this Ground Truth study was to understand how young Kenyans without disabilities conceptualized disability, how they view people with disabilities in their communities and beyond, what interactions they routinely have with them and how AT relate to their own vision of disability.

To this end, a series of focus groups, with young people, both males and females, between the age of 18 and 26 were carried out in different regions of Kenya. Study details were shared among Shujaaz fans on social media and other media platforms run by the organization. Snowball sampling techniques were also used, with young people invited to recommend friends for participation if they wished to do so. Volunteers were screened for age, gender (as we aimed to maintain a balance between the genders of recruited participants in each group) and geographical location. Our aim was to conduct at least one focus group in each of the following regions of Kenya: Nairobi, Central region, Western Region, Rift Valley, Nyanza and Coast Region. These areas were selected as, based on the 2007 Kenya National Survey on Persons with Disabilities, they provided a good representation of areas with different prevalence of disability and various levels of socio-economic status. We did not screen participants for their degree of relationship with people with disabilities, as we aimed to recruit a group of individuals who offered a natural representation of local youth without introducing artificial quota based on familiarity with disability. If they met the selection criteria and agreed to take part in this study, participants were offered a small compensation to cover any expense that they incurred (such as transport to the focus group venue or loss of work time). In total, 7 focus groups were conducted in the following regions: Mombasa (9 participants, 5M and 4F), Dandora (8 participants, 3M and 5F), Kilifi (7 participants, 4M and 3F), Chavakali (7 participants, 2M and 5F), Nyeri (7 participants, 3M and 4F), Iten (8 participants, 3M and 5F) and Bondo (8 participants, 5M and 3F). Focus groups featured a mixture of group discussions and interactive activities to explore how young participants described and defined disability, what types of interactions they had with people with disabilities, how comfortable they feel interacting with people with disabilities in different roles (such as teachers, doctors, friends and potential romantic partners), and what attitudes and beliefs about disability were promoted and encouraged in their communities. Questions and activities were also focused on assessing awareness of AT and understanding how AT influenced youth’s idea of disability both positively and negatively. These aspects were explored both directly and indirectly. For example, participants were directly asked what tools and technologies they knew that could facilitate everyday tasks for people with disabilities and what they think about them. At the same time, when asked to describe a person with disability, participants often included assistive technology in the portrait or description. If that was the case, the moderator would ask additional questions about why the assistive technology was present in the description, what function it served and what would be the consequences if the person with disabilities did not have access to it.

Before the start of the focus groups, for all participants, the purpose of the research was explained and verbal consent for data collection and analysis was obtained. Focus groups were carried out in Kiswahili and Sheng Slang, a language that mixes Kiswahili and English, which is very common among young people in Kenya. Ethics approval for secondary data analysis was obtained through the UCL Research Ethics Committee.

#### 2.2.2. Data Analysis

Focus group audio recording underwent verbatim transcription and was subsequently translated from Sheng to English. Researchers at Shujaaz Inc. who conducted focus groups oversaw the translation to ensure that the original meaning of conversations was retained. Translated focus groups transcripts were then analyzed using an inductive approach to thematic analysis, leveraging investigating both semantic and latent meaning of conversations [54,55]. The aim of the analysis was to develop a comprehensive understanding of how young participants conceptualized disability, identifying the factors that influenced their attitudes and beliefs towards people with disabilities both positively and negatively, and assessing the role that AT plays in shaping their perception of disability.

Initial coding was carried out by the first author and initially confirmed by the fourth author. As the analysis progressed, discussions with the wider team were regularly carried out to ensure that the themes conceptualized provided an accurate and comprehensive responses to the above research questions.

### 2.3. Study 2: Lived Experiences of Stigma and AT Use among Kenyans with Disabilities and Their Allies

#### 2.3.1. Participants and Procedures

The secondary data used for this study derived from the planning and consultation phase of the UK FCDO funded Overcoming Stigma toward Assistive Technology project (OSAT), itself part of the wider AT2030 consortium, led by the Global Disability Innovation Hub. The OSAT project tests strategies to overcome stigma toward the use of assistive technology in Kenya. Prior to research design, a two-phase planning process was conducted, whereby we sought the expertise of two distinct stakeholder groups.

Firstly, we held a consultation meeting with representatives of ten Kenyan Organizations of Persons with Disabilities. This comprised an unstructured discussion of the representatives’ views regarding key issues concerning disability stigma and discrimination in Kenya, particularly with respect to assistive technology. The discussion lasted for 1 h and was audio-recorded with participants’ consent. The aim of this first consultation was to get a macro-level perspective on disability stigma and assistive technology. Second, we consulted with six Kenyan people with disabilities who were assistive technology users and six of their friends without disabilities. Of the six participants with disabilities: two had hearing impairments, two had physical impairments and two had visual impairments, and they used the following assistive devices: hearing aids, wheelchairs and white cane. Three of the people with disabilities and two of the people without disabilities interviewed were male. Each pair was interviewed separately and responded to a set list of questions about life with a disability and assistive product and about their friendship more generally. Interviews were video-recorded. The aim of these second consultations was to understand the content of disability stigma and discrimination in Kenya from the perspective of both people with and without disabilities.

The primary purpose of these consultations was to contribute to the research design of subsequent project studies (e.g., ensuring that research surveys contained measures that assessed the key issues raised by the consultations). However, we were also interested in what individuals involved in these consultations thought about disability stigma and discrimination and assistive technology. Participants in each round of consultations were purposively selected to represent a range of perspectives and viewpoints. Specifically, we sought out both representatives of key Organizations of Persons with Disabilities (OPDs) working in Kenya and individuals with a range of impairments who utilized assistive technology. Moreover, we also purposively consulted the friends without disabilities of participants with disabilities in order to understand how AT may (or may not) influence wider social contexts.

#### 2.3.2. Data Analysis

We conducted thematic analysis [54] on the corpus of data derived from the consultations to gain a deeper understanding of the lived experiences of stigma experienced by people with disabilities in Kenya as well as perspectives from their allies, by finding repeated patterns of meaning, and going beyond semantics to uncover underlying ideas, assumptions and conceptualizations [54] about disability stigma and perceptions around assistive products. All transcripts were coded by Author 3, and checked by Author 2. The codes, and their associated extracts, were then examined by Author 3 and themes synthesized, which were then discussed as a wider team.

## 3. Results

As both studies sought to address two distinct research questions, we present findings from each study in relation to each research question separately, with the aim of highlighting both differences and similarities between points of view of Kenyans with and without disabilities.

### 3.1. Factors Shaping Disability Stigma

#### 3.1.1. Study 1

Most of the elements that influenced both positive and negative attitudes surrounding people with disabilities are linked to mental images and associations about disability that are made by Kenyan youth without disabilities sometimes in absence of real-world interactions. Many of these associations are linked to widespread societal beliefs, personal impressions that are associated with exposure to positive and negative models. The six themes identified were: What is disability? Societal influences, religion and spiritual beliefs, disability as punishment, one of them and one of us, confirming and defying stereotypes and access to resources.

##### What Is Disability?

One of the main factors that influenced how young participants perceived and related themselves to people with disabilities was how they defined and conceptualized disability in the first place. Unfortunately, for the majority of youth, disability was automatically associated with a lack of capacity that makes one needier than a person without disabilities and, potentially, a burden on others.

“A disabled person faces many challenges. Let’s say going for nature calls, he has to be carried, he can’t wash, he can’t cook so he was dependent on neighbors and friends to bring him food”

Iten group—P3

Language plays a huge part in shaping these perceptions. Across all seven focus groups, when initially asked to describe a person with disabilities, all participants did so using a series of negative images of what a person with disability would, in their opinion, be unable to do unless supported by others.

“We look at disability as someone who cannot manage themselves, but they can [only] do something through the help of people”

Bondo group—P7

This perceived inability makes any relationship with a person with disability unbalanced and youth were concerned that having a partner or a family member with disability might create further challenges and difficulties for themselves.

“If my child turns out to be disabled, I know I will feel [sorry about] it because he/she will go through a lot, there are challenges that he/she will face. Like, when I go to work, I would have to tend to the child before I go out. So, there are some issues that I would have to leave pending, so that I can tend to the child and then go my way”

Mombasa group—P2

Persons with disabilities were often seen as unable to provide substantial contributions to society and several participants did not feel that they could perform most jobs or occupy positions where they would have to provide help and support to others. For this reason, several young people stated that they would prefer not to have people with disabilities occupy roles such as doctors, teachers or even landlords.

“What I know is that a doctor should be physically fit, when he/she hears of an emergency he goes”

Iten group—P5

On the other hand, participants who described disability mainly as a societal or situational construct, more akin to the social model or a critical view of disability, were much more likely to exhibit better attitudes towards people with disabilities. These participants recognized that disabling interactions often occurred as a result not only of bodily limitation but also of social and situational factors and that people without disabilities had responsibility in their creation.

“Disability is about discrimination”

Chavakali group—P4

“If he [a person with a disability] came with an intention of joining us, our activities will have to change so that he feels involved depending on his disability. If we one is deaf you can just write down thing and he can join”

Nyeri group—P1

##### Societal Influences, Religion and Spiritual Beliefs

Most of the young people who participated in the focus group conducted across all various regions of Kenya had strong religious and spiritual beliefs and reported a strong sense of belonging and trust in the views of their communities. As awareness of disability could be very poor in some communities, this could lead to very high levels of stigma in some cases. One of the most damaging incorrect beliefs was that a person could acquire a disability as a result of a curse. Despite the overall tendency to attribute increased superstitious beliefs about disability to people living in rural and remote areas, we noticed at least a few participants in every of our focus groups who believed that a person could acquire a disability as a result of a curse. In some cases, young people stated that a person bewitching another could cause them to have an accident that would result in disability. However, others thought that a curse, usually cast by someone who sought to take advantage of the individual or who wanted revenge for some reason, could simply lead to the sudden onset of an impairment.

“It is usually jealousy because you can find that someone just hates you, so they consult a witch and then a spell is cast on you that may make something bad happen to you like an accident. Sometimes someone loses consciousness, so he will be disabled”

Bondo group—P1

Interestingly, disability as a curse was more often associated with cognitive impairments and blindness rather than physical or hearing impairments. Regardless of the reason behind the spell, punishment or curse, when youth believed that a person had acquired a disability in this manner, they held very high levels of stigma and they were unlikely to want to associate with the person with disability. Most of the times this was because of fear as they assumed that the curse could be somehow passed on to them.

“I will go for the first four [people who acquired their disability as a result of accidents or diseases] and I will help them, but the other ones like the ones getting cursed, the community thinks if you help the cursed ones the curse will befall you too. Because you don’t know why they were cursed”

Kilifi group—P5

In contrast, positive community attitudes could lead not only to much lower levels of stigma and increased acceptance, but also proactive efforts towards inclusion. For example, in the rural town of Nyeri, elders and other influential members actively promoted positive community attitudes towards people with disabilities and encouraged others to support them and ensure they had access to education and other essential services.

“Where I am from there is nothing like that [referring to negative behaviour towards people with disabilities”. They [the elders] love them so much. And if you do something wrong to them [and] an old woman notices like discrimination, you will know that the heavens judge and we live on the same earth. They [the elders] encourage us not to hide them, and they [people with disabilities] should be taken to special schools so that they can get helped”

Nyeri group—P2

Religion was also generally associated with positive attitudes and supporting behavior towards people with disabilities. Many young participants who had strong religious beliefs stated that disability status should not matter in how you would relate to another person as all fellow humans should be treated with kindness and respect.

“For example, I have found one [a person with disability] with luggage and helped them carry it to their destination and they appreciate it. The Bible states that a day should not end without someone being grateful for something you have done to them”

Kilifi group—P3

##### Disability as Punishment

One association that some of our young participants made about disability, which always led to negative views of disability, was the fact that it could occur as a just consequence of reckless, unlawful or immoral behavior. The most common example cited by youth who lived in an urban environment was the acquisition of a cognitive impairment as a result of drug use (particularly khat).

“There are many young people who smoke khat in our community. That is something I have seen happening and these persons who are using drugs, for example khat or bhang, have been warned on several occasions but he did no heed to this and then they go mad”

Mombasa group—P2

Road traffic accidents were also a common cause of physical impairments due to amputation or spinal cord injury, but young people specifically mentioned that drunk or foolhardy drivers who ended up in an accident, acquiring a disability because of their own wrong choices. In some rural areas, an amputation could even be inflicted as a punishment for a crime, such as stealing. Although, in these cases, young people stated that the punishment was too harsh, they still maintained that the thief had a measure of responsibility in the onset of their own disability

“If you go and take someone’s property you might get caught, then maybe you might get caught or a part of the body is cut, maybe your leg and that will make you end up in a wheelchair. It’s terrible when they do that, but people know they should not be stealing”

Bondo group—P8

Finally, disability could also be caused as a result of a “divine or spiritual” punishment that would befall either the individual or, in some cases, their child.

“Or it is like, maybe laughing at someone who has a particular disability, maybe I broke my arm and you laughed at me. So then in that process, maybe you give birth to a child with the same situation as me, so they may say that God cursed him”

Chavakali group—P7

In all those instances, participants felt much less inclined to offer empathy or support to the person with disability, as they considered the impairment a punishment for their wrong behavior. Although they might feel bad for any hardship faced by the person with disabilities, hardship was considered a part of most people life and those who experienced it due to circumstances outside their own control should be offered more support compared to those who might have “brought it upon themselves”. Kilifi group—P3.

##### One of Them or One of Us

A key factor that influenced how youth perceived people with disabilities is how similar, or different, they considered people with disabilities to be from themselves and their communities. Unfortunately, for many young participants, disability was seen as something that would make people different from others, automatically turning someone into an outcast. This difference was often associated with the physical appearance of more visible disabilities, but it could also be due to the behavior of someone with a cognitive impairment or the different mode of communication of a deaf individual using sign language.

“[Disability is] The situation where someone can lack an organ or a part of him that make him look different from others”

Dandora group—P2

“People who are mad don’t behave like anyone else, you can see very quickly that they are different”

Kilifi group—P1

Many young people were afraid that being close with someone with a disability could negatively reflect on them and cause them to be excluded from society by association. Especially when it came to thinking about having a boyfriend or a girlfriend with a physical disability, participants felt shame and fear at being negatively judged by their peers.

“If this girl has a disabled boyfriend, or maybe this boy who uses a wheelchair, these other girls, not all of them will have the positive side, there are those who will tell you all sorts of things. You see, even if you love him, that will make this girl feel low and in that process this girl will be looking for all sorts of blame games so that they can separate”

Nyeri group—P6

Finally, even when young participants saw people with disabilities engaging in normal everyday activities such as going to school or participating in some community groups, these were often separate from the ones they attended, which reinforced the beliefs that young people with and without disabilities had no shared experience.

“People with disabilities go to school in Kibarani. They have a different school for the disabled there, a place for the deaf… they also have a teacher there who is blind”

Kilifi group—P6

Unsurprisingly, in exact opposition, young participants who had experienced frequent positive interactions with people with disabilities were the least likely to hold negative stigmatizing beliefs. In most cases, these interactions took place in schools or youth groups that participants attended, which confirmed that people with disabilities could be well integrated within their own communities. Young people who had friends with disabilities in school or youth groups were often the first to point out interests and experiences that they had in common rather than focusing on differences.

“I learnt in Iten primary that behind it [the participant’s school] there is that school of the deaf, so we used to get here to pass time. They are just like us. I do not see any difference with us, it is just that we are….”

Iten group—P7

Although participants acknowledged that the person with disability might have a different capabilities or be faced with greater challenges in carrying out certain tasks, they did not feel that they should be shielded, pitied or treated differently.

“This guy in my boarding school doesn’t have legs, so he has his own seat there […] Then he doesn’t have fingers to hold a pen. So, he uses one finger and uses it to write. And then he is pretty sly, just the other day he even stole my pen, but when he stole my pen, I also gave him a knock on his head. He is just a human being as I am so I it’s tit for tat, he stole my pen, and I gave him a knock on his head”

Mombasa group—P4

##### Confirming and Defying Stereotypes

As disability was often associated with inability to provide for oneself and being rejected by society, negative images and interactions that reinforced these stereotypes created a positive feedback loop that substantially increased stigma. Most young people talked about interactions with negative connotations taking places in marketplaces, junkyards and street sides where people with disabilities were seen begging. Feelings of young participants were split between pity and suspicion, as some reported feeling sorry for people with disabilities, whereas others were concerned that people could fake their disability or leverage it in order to avoid the need to work.

“They borrow money, their business is to borrow money”

Dandora group—P7

“There are some who use their disability to make some money”

Chakavali group—P2

This duality between pity and suspicion or fear was even more pronounced towards people with learning and cognitive disabilities, who were either described as angry and violent or as simpletons who could not provide for themselves or behave like a “regular” person.

“He [referring to a person with cognitive disabilities] is like a mad person, like he/she is grown but still plays with children like an older person just playing with kids.”

Iten group—P1

“Those who are mentally challenged, they might hit you for just looking at them. I was pushed and insulted while excusing them to pass on a muddy road [sic]”

—Kilifi group P5

But people with cognitive and learning disabilities were not the only ones described as angry and aggressive. Some young people felt particularly resentful towards all people with disabilities as they believed they took advantage of their disability to avoid repercussions when behaving abusively.

“You know these disabled people, they like mocking people and they are rude, and they do that because they know that no one can beat them up, like no one can spank your butt”

—Mombasa group P4

On the other hand, interactions, even superficial ones, with people with disabilities that did not fit these stereotypical images often led to more positive attitudes towards people with disabilities in general. In some cases, these were linked to the visibility of public figures such as president Kibaki who, in 2002, was using a wheelchair during his swearing in ceremony as he had recently been involved in a road traffic accident. However, in most cases, young people were more interested in more “low-profile” role models that they could relate to, especially people with disabilities that were successful at their job or who could run a profitable hustle.

“Yes, we have seen them on T.V, in the news [thre was] a woman writing using her hand. Like she has no fingers. She has legs but can’t walk and she has toes. She knows how to draw, she’s such a big artist”

Mombasa group—P2

“There is a person whose leg starts from here until somewhere here, there are no hands but there is something special that he has and when he talks. And he talks to large crowds”

—Dandora group—P8

People with disabilities who were seen as successful in their work were regarded with particular respect as young people thought that they would have had to show above average competence to obtain their position as they were likely to have faced greater challenges. Furthermore, some young participants felt that people with disabilities might exhibit greater empathy to others, making them more adept to mentorship and caring roles.

“A doctor who is lame then gets a patient who is lame can advise the patient on how he got there, yet he is [also] lame so he should not take it as a way that will drain his life”

Iten group—P5

People affected by albinism were often cited as an example of this as young people classified them as people with disabilities, yet they also knew many of them who were successful business people and active members of their communities.

“Let me say something, where I am from. We have an albino who has an M-Pesa shop and we were told that theirs is a skin condition, so we don’t think of him as a disable person. But we do know they have a disability”

Bondo group—P3

##### Access to Resources

For many of the young people who took part in our focus groups, the worst implications of disability were related to the perceived limited ability to do work and slip in the ever-present trap of poverty. When disability was accompanied by access to resources, especially financial resources, its impact was seen as a lot less severe and the stigma attached to it was also significantly lower. People who had access to greater financial resources were seen as ‘less disabled’ either because they could have access to tools, or support that would reduce their own impairment or simply because they could escape poverty.

“There are bosses that are disabled, but have their cars there which they drive to work, so there’s no problem there”

Kilifi group—P5

“Many people are suffering because they have no cash, they do not have anyone who can help them. But somebody like the president has money, even right now if anything happens to him, he can help himself but not people like us”

Dandora group—P2

Even when asked about the interactions that they were willing to have with persons with disabilities, participants stated that they were more willing to associate with people with disabilities that they perceived as more resourceful, either economically or personally, as they felt they brought more to the relationship.

“If two boys, a deaf and this one [who] is not deaf both have vibed me (courted me), I would accept the one that can hear. But if the deaf boy has money and he is nice I would accept him because he can help take care of me”

—Chavakali group P3

“When I was in class 7 I had a disabled desk mate, he was a good friend and was good at Maths. We were classmates and he helped a lot of people who knew him, so we interacted as usual”

—Mombasa group P1

#### 3.1.2. Study 2

Three themes were identified that broadly mapped onto the themes identified through analysis of the perspectives offered by youth without disabilities in Study 1. These were traditional and religious views of disability, lack of understanding, and lack of accommodation and invisibility.

##### Traditional and Religious Views of Disability

This theme encompassed participants’ view that the many Kenyans without disabilities adopt traditional views of disability and the perception that this creates false narratives about people with disabilities and disability itself. For example, one participant noted that Africans commonly hold traditional religious beliefs about ancestral curses being the cause of disability:

“We [are] Africans, so whenever your parents give birth to someone who is disabled, they think it is like you are cursed… they think you are [a] cursed one”

—Physically disabled male video participant

Other participants noted how interpretations of disability as having a religious cause can precipitate stigma and discrimination:

“While my friend is pushing me, they sympathize… [they say] sorry for what happened, we pray for you so that one day you will walk. But to me, I appreciate how God made me”

—Physically disabled male video participant

This excerpt also demonstrates how Kenyans with and without disabilities may draw different conclusions from a religious interpretation of disability. For some Kenyans without disabilities, a disability is an object of pity and if from God must therefore be a curse, whereas for some Kenyans with disabilities, an impairment can be viewed as something positive given by God.

##### Lack of Understanding

Another aspect of stigma bought out in the consultations were encounters where Kenyans with disabilities experienced rejection and avoidance. Participants observed that Kenyans without disabilities commonly seek to avoid people with disabilities, for example seeing their impairments as contagious, as shown in the example below:

When I enter into a matatu, people think I’m sick, most of them move away from me”

—Hearing impaired female video participant

Even where Kenyans without disabilities do not adhere to the belief that disability is contagious, they may seek to avoid interactions with Kenyans with disabilities, due to generalized negative attitudes about impairments as “different”. Below, one participant reflects:

“I used to fear the disabilities, I’m sorry to say though. I didn’t want to have interactions with them, I didn’t want to get so close, [it] felt like something different to me… I had some fear in me, so I kept my distance”

—Participant without disabilities

As the participant suggests through his use of past tense, these attitudes are malleable. In this case, the participant became more comfortable with people with disabilities through starting a friendship with a person with disabilities.

##### Lack of Accommodation and Invisibility

The built environment and societal structures are developed with people without disabilities in mind and our research suggests that this lack of accommodation shapes the experiences of stigma and discrimination for Kenyans with disabilities. One participant remarked on their experience of exclusion in the workplace:

“At the workplace… most of the colleagues are [not] hearing [impaired], actually the majority [of them]. They have their own cocoon; they have their own groups… I was alone, no one was able to talk to me because of the issue of sign language”

—Hearing impaired, video participant

This example illustrates that effort is rarely made to fully involve people with disabilities, for example through overcoming communication barriers via learning sign language. Often, unwillingness to accommodate people with disabilities prevents them from accessing opportunities on an equitable basis to people without disabilities:

“There was a time there was an advert for a job, I applied for that job, I was shortlisted, I was called for the interview… I was ushered into the room, so one of the panellists saw my hearing aid, they were like what do we do? There is no SL interpreter would he be able to write?”

—Hearing impaired video participant

Even where Kenyans without disabilities have the opportunity and ability to communicate with Kenyans with disabilities, interactions may be avoided, and this can lead to Kenyans with disabilities feeling overlooked:

“When they see a wheelchair, they’ve not even seen me, they’ve not interacted with me… simply because they judged me because I’m a wheelchair user. That is a stigma”

—Physically impaired, female video participant

At other points, the use of assistive devices can trigger invasive encounters with Kenyans without disabilities:

Even those who are grownups are following you, wanting to know…what is this cane for? They try to grab it”

—Visually impaired female video participant

In this example, it is not the person with a disability who is invisible, but rather their preferences and privacy. Specifically, their assistive device is treated not as something that is a private possession, but a piece of public property to be touched and grabbed.

### 3.2. Role of AT in Disability Stigma

#### 3.2.1. Study 1

Overall, the knowledge that young participants had of assistive technology was limited and mainly concerned with mobility aids for people with physical or visual impairments (e.g., wheelchair, crutches and white canes). Assistive technology was sometimes described as a visible identifier of disability. However, most young people saw it in a positive light as a tool that could enable people with disabilities to be more active and independent. The two themes that we conceptualized were: AT as a disability identifier and AT as an enabler.

##### AT as a Disability Identifier

For many youths, AT would often represent a visible marker of disability. When asked to draw or describe a person with disabilities, participants often pictured people with walking impairments who used wheelchairs, crutches or walking sticks for mobility. Even visually impaired people were depicted using a white cane, as these assistive devices were all visible and easy to identify, indicating that participants used these visible indicators to categorize people with disabilities. Interestingly, young people often used the assistive device itself, rather than the impairment, to label the type of disability that they attributed to an individual.

Moderator: “What is the disability of this person?”

P1 “He has crutches”

Moderator “What about this one? Who drew this?”

P4 “I did it. That one uses a stick to walk because he has only one leg” Kilifi group

Across all focus groups, participants almost exclusively mentioned simple assistive devices that were linked to mobility and only associated them with people with physical or visual impairment.

##### AT as an Enabler

In most cases, participants saw AT in a very positive light and described it as a valuable tool that could help people with disabilities carry out their daily activities and be more independent. Not having access to assistive technology would compound the limitations that were associated with disability and negatively impact the opportunities available to people with disabilities. For example, P6 from the Dandora group stated that people with walking impairments could always use wheelchairs to move around, but if they did not have access to them, they had no alternative to having to crawl on the ground which was seen as an undignified way to move and would restrict where people could go and what they could do in their communities. Young people saw assistive technology as an essential enabler that allowed people with disabilities to access opportunities for work education and social life.

“I schooled with him [a young boy with disabilities] when he was that way, and he even had his own chair that was designed for him. So that’s a special chair that was made for him that enabled him to sit on his stomach and he was in boarding school”

Mombasa group—P4

Participants had heard of more technologically complex assistive devices such as motorized wheelchairs, adapted cars and robotic devices, but these were seen as only available to people in the Global North or extremely wealthy people with disabilities. Access to advanced assistive devices, combined with a higher economic status, could completely erase any disadvantage brought by disability, so much so that any impairment the person had did not really matter anymore.

“Most of them [people with access to high tech devices] are of high calibre, they are out of reach, whether disabled or not. There are bosses that are disabled but have their cars there which they drive to work, so there’s no problem there”

Nyeri group—P5

#### 3.2.2. Study 2

Like Study 1, participants with disabilities and their allies viewed AT in a positive light as a tool for independence and empowerment. However, they were also cognizant of the ways that assistive technology use can attract stigma.

##### AT as a Beacon for Stigma

Some participants with disabilities felt that assistive devices attracted stigma from Kenyans without disabilities. For some participants, this meant avoiding using assistive devices in public for extended periods of time, despite the health problems and difficulty caused:

“She needs to use a calliper… [she] hasn’t used it for 5 years due to pity people have… When you’re limping, it is not taken as seriously…[there is] more ability to blend in in the community without use of [the]crutch. I know it’s having an adverse effect on her health”

—Female OPD consultee with disabilities

Others, particularly those reliant on assistive devices to get around, may instead choose to isolate themselves at home due to fear of stigma, as evidenced below:

“I have a friend who has a prosthetic leg, she many a times avoids walking into the busy Nairobi streets, people stare at her, maybe laugh when they see her use her leg… Many a times [she] avoids…walking out. She keeps to herself, if [there is] something she needs, she’d rather send somebody than go for it herself”

—Female OPD consultee with disabilities

In sum, the experiences of our participants suggested that, in some cases, assistive technology use can attract stigma.

##### AT as an Extension of the Self

Several participants with disabilities saw their assistive devices as an extension of self and found that the devices were instrumental in bypassing areas of functional difficulty, for example, moving around safely in the built environment:

I can perceive for example an aeroplane flying over me, motorbikes, cars passing, so when “I’m walking on the road, I am able to perceive these sounds and can move, so I am not hit by a vehicle”

—Hearing impaired female video participant

Some Kenyans with disabilities viewed their assistive device as a literal replacement or stand in for an area of functioning. The example below is taken from a participant who uses a white cane. The participant describes the assistive device as taking on the role of being her eyes and views it as a substitute for her visual impairment.

“White cane changed my life… it helps me like how those people who sees, they see with their eyes and it is like my eyes… The white cane helps me to walk along…where there is trees, stones, it helps me”

—Visually impaired female video participant

The ability of assistive devices to alter the self was also recognized by Kenyans without disabilities, who, in some cases, thought an assistive device was able to compensate for disability to the extent of eliminating it:

“[Her AT]…has ameliorated the effect of disability…now she is able to interact with all people, those hearing and not hearing…those devices, [they] promote efficiency and performance”

—Female video participant without disabilities

##### Independence and Empowerment

Participants with disabilities described a range of personal benefits associated with the use of assistive devices. Many participants explained how assistive products made them feel more self-positive and described several activities they are able to enjoy through using assistive products.

“It helps me to be confident, many times, I segregated myself because I feel insecure. [I am] able to face my friends, able to see my friends”

—Visually impaired female video participant

“It’s given me dignity… I’m able to do my daily activity as a human being…I can interact with people socially…”

—Physically impaired female video participant

“I am able to enjoy music with the assistive device, I use my earphones, I can dance and enjoy music”

—Hearing impaired male video participant

Some Kenyans with disabilities, especially those with physical and visual disabilities, felt a sense of independence and empowerment through their use of assistive devices.

## 4. Discussion

This paper presented two separate studies which sought to explore the factors that shape stigmatizing attitudes, beliefs and interactions about disability among Kenyans with and without disabilities. Furthermore, we specifically looked at how participants viewed AT and what role they played in the construction of disability stigma. When comparing findings from the two studies, we noticed both significant overlaps and striking contrast between the perspectives of people with and without disabilities. Furthermore, most of the factors we have identified as affecting the interactions between Kenyans with and without disabilities from the emergent themes highlight a series of tensions that stretch on a continuous spectrum rather than having merely positive or negative connotations.

For example, the influence of traditional, religious or other spiritual and societal beliefs was found to have both positive and negative implications, depending on the context. On the one hand, beliefs that disability could occur as a result of a curse had severe negative implications that led to avoidance and rejection of people with disabilities. This was often due to the fear of curses being transferred or the perception that a curse would have befallen only someone who deserved it in the first place. This was in line with the findings presented by [3,56], who highlighted how traditional societal beliefs linking disability to a curse or witchcraft were linked to negative behaviors towards people with disabilities and their families that ranged from exclusion to violence. Attributing one’s disability to God’s will, however, was not necessarily linked to negative attitudes. Among both people with and without disabilities, this religious belief could be associated with increased levels of acceptance, as regardless of differences, all humans were related to God, and pride in one’s identity as one’s disability was a gift from God. These contrasting views can help to merge the conflict observed in some previous literature looking at the influences of religion on the perception of disability in Sub-Saharan Africa. In this context, our findings justify both the viewpoint of authors such as [57], who highlighted how traditional and religious beliefs are often leveraged to perpetrate oppression against people with disabilities in Nigeria, and [58], who argued that religion could play a key role in engaging communities in questioning ethical and unethical assumptions, or [59], who pointed out the supporting and advocating role that religious communities can play for individuals with disabilities and their families.

As previously shown in relevant literature [60,61], findings from both studies confirm that the occurrence of frequent positive and normalized interactions between people with and without disabilities had a significant impact on minimizing disability stigma. When people without disability had no opportunity to interact with their peers with disabilities, they were more likely to see them as different, not part of their societies, and were more likely to fear, pity or simply avoid them. This persistent and constant exclusion, which occasionally alternates with violation of privacy and personal spaces caused by the curiosity of a passerby who reaches out to grab one’s assistive technology as reported by participants in Study 2, leads not only to loss of opportunities, but also disempowerment, resentment and feelings of helplessness. When these feelings lead people with disabilities to adopt aggressive behaviors or simply call out ableist episodes, as seen from some the comments of young people in Study 1, this reinforces negative stereotypes and further damages intergroup relationships [62]. In contrast, participants without disabilities who had frequent interactions with peers with disabilities displayed more positive attitudes and were more likely to consider people with disabilities as part of their own social circle. It is important to notice that, as suggested by the statement from one of the allies of people with disabilities who was interviewed in Study 2, fears and negative beliefs around disability are not immutable and can be radically changed by interactions that help to subvert stereotypes [29,63].

The negative association between disability and the inability to work and provide for oneself was one of the most pervasive held by participants without disabilities and it was reflected in the experiences of exclusion and discrimination reported by participants with disabilities. This is unsurprising, as it is an incredibly common stereotype which has been reported by several studies irrespective of the geographical region in which they were carried out [31,64,65]. As expected, this also led to the fact that positive images of people with disabilities in the community, who were successful workers and hustlers, had a strong positive impact on the perceptions of people without disabilities. More interestingly, this also created a dualist perspective in the minds of people without disabilities between disability and the availability of resources, seen as financial, personal or social capital. The relationship between poverty and disability is not a new one and the knock-on effect between these two factors has been documented by several authors [53,66]. However, a unique insight that transpired from the stories shared by young people without disabilities in Study 1 was that the other person’s perceived availability of resources played a bigger role than disability status in many of their interactions. Although this might appear surprising in light of the strong cultural and religious beliefs of many participants, it is not unexpected, as youth in the Global South often live under the threat of poverty and are heavily subjected to neoliberal and capitalistic narratives that reinforce this transactional mentality [67,68].

The relationship between disability stigma and the perception of ATs showed some interesting discrepancies between participants with and without disabilities. Youth knowledge of AT in Study 1 was relatively low, with awareness largely limited to mobility devices for people with physical and visual impairments. However, their perception of AT was largely positive. On the one hand, AT was perceived as an easy identifier of disability that helped them to label individuals and potentially adjust behaviors in various interactions. At the same time, ATs were seen as facilitating independence and as an empowering tool for people with disabilities. Independence and empowerment can be understood as closely interrelated concepts encompassing functioning and affect, respectively [69]. That is, participants in Study 2 reported both instances where they could functionally complete tasks (e.g., listening to music) with AT without having to rely on others (i.e., independence) as well as positive affect (e.g., dignity) experience as a result of being independent (i.e., empowerment). Moreover, access to AT was linked to the perception of increased available resources, both as an identifier of wealth but also as a capability enhancer. The stories shared by people with disabilities in Study 2 showed a more nuanced perspective on AT that incorporated both positive and negative aspects. The perception of ATs as a tool that enhanced capabilities was definitely present, and it was also accompanied by an enhanced sense of embodiment akin to the one reported by participants in [46,70]. However, the role of ATs as identifiers of disability was seen as a lot more problematic as participants found that visible devices prevented them from blending in the crowd if they wished to do so [38], attracting unwanted attention and stigma [45], and even overshadowing their own identities [46].

Overall, these results have important implications for the development of policies, campaigns and other initiatives that aim to reduce stigma and promote the inclusion of people with disabilities. First, the findings highlight that stigma is a common experience for Kenyans with disabilities and that, despite its benefits, the danger of experiencing stigma and discrimination may influence some Kenyans with disabilities to avoid assistive devices, despite the consequent health and social costs. As such, effective interventions to ameliorate disability stigma and discrimination warrant urgent consideration by policy makers, including within initiatives scaling up the provision of assistive technology.

Second, with respect to which interventions may be effective, our findings are also instructive. For example, as one of the main factors in improving the quality of interactions between people with and without disability was the occurrence of frequent intergroup contact and cross-group friendship, especially at a young age, it is clear that these policies and initiatives that promote encounters are likely to be highly successful [71]. This integration can be promoted at the national level through the furthering of efforts towards inclusive education, but also at the local level by promoting inclusive events for children, potentially in relation to religious institutions who have shown to be powerful influencers.

Similarly, considering the importance that young people, and people with disabilities, attribute to the ability to work, be productive and provide for oneself and their family, it is essential for future initiatives to focus more on providing better opportunities for people with disabilities to enter and be part of the workforce, and for campaigns to showcase the professional success of local people with disabilities who can serve both as a role model and myth buster. Finally, more attention should also be dedicated to the portrayal of ATs to inform people about their usefulness, but also promote a more realistic image of them that goes beyond common stereotypes that sees them as universal problem-solvers without which people with disabilities would be completely helpless [39]. ATs are a key tool for millions of people with disabilities worldwide, but they are a tool that require skills to be used, cannot bridge gaps created by fully inaccessible environments and should never overshadow the identity or pre-existing capabilities of the individual.

### Limitations

There were a few limitations of note in both studies. In Study 1, all participants were young Kenyans between the age of 18 and 26. Although the national median age in Kenya is 20.1, indicating an overall young population, older people might hold different views of disability in light of having been exposed to different cultural influencers. In Study 2, participants were people with disabilities who were assistive technology users, thus they might conceivably hold more positive attitudes to AT relative to those who need AT but do not use it (e.g., due to stigma). Moreover, we spoke to six allies without disabilities who likely have more positive attitudes to disability in general in light of their contact experiences and familiarity with a friend who has a disability. The data in Study 2 also comprised secondary data analysis of consultations to inform a later project intervention, rather than dedicated qualitative research designed to address the subject under study. As such, it is possible that some of the nuances of participant experiences of stigma were missed by our line of empirical inquiry.

## 5. Conclusions

In this paper, we present two studies looking at the factors that shape the development and the experience of stigma surrounding disability and AT from the perspective of Kenyans with and without disabilities. Our results show that disability stigma is shaped by factors ranging from the conceptualization of the causes of disability, to societal and religious beliefs of the community, misconceptions regarding the ability of people with disabilities to work and be productive, inaccessible environments and lack of opportunities for positive intergroup exchanges. ATs are generally seen as important enabling tools. However, they also work as a constant visible mark of disability that can attract unwanted attention and occasionally be seen as more important than the person with disability who is using them.

## Data Availability

The data presented in this study could be made available on request from the corresponding author according to the ethics and data protection guidelines of University College London. The data are not publicly available in order to protect privacy of participants.
